# Effects of Glucono-δ-Lactone and Transglutaminase on the Physicochemical and Textural Properties of Plant-Based Meat Patty

**DOI:** 10.3390/foods11213337

**Published:** 2022-10-24

**Authors:** Haesanna Kim, Mi-Yeon Lee, Jiseon Lee, Yeon-Ji Jo, Mi-Jung Choi

**Affiliations:** 1 Department of Food Science and Biotechnology of Animal Resources, Konkuk University, Seoul 05029, Korea; 2 Department of Food Processing and Distribution, Gangneung-Wonju National University, Gangneung-si 25457, Korea

**Keywords:** textured vegetable protein, binder, water holding capacity, texture analysis, sensory test

## Abstract

Due to growing interest in health and sustainability, the demand for replacing animal-based ingredients with more sustainable alternatives has increased. Many studies have been conducted on plant-based meat, but only a few have investigated the effect of adding a suitable binder to plant-based meat to enhance meat texture. Thus, this study investigated the effects of the addition of transglutaminase (TG) and glucono-δ-lactone (GdL) on the physicochemical, textural, and sensory characteristics of plant-based ground meat products. The addition of a high quantity of GdL(G10T0) had an effect on the decrease in lightness (L* 58.98) and the increase in redness (a* 3.62). TG and GdL also decreased in terms of cooking loss (CL) and water holding capacity (WHC) of PBMPs. G5T5 showed the lowest CL (3.8%), while G3T7 showed the lowest WHC (86.02%). The mechanical properties also confirmed that G3T7-added patties have significantly high hardness (25.49 N), springiness (3.7 mm), gumminess (15.99 N), and chewiness (57.76 mJ). The improved textural properties can compensate for the chewability of PBMPs. Although the overall preference for improved hardness was not high compared to the control in the sensory test, these results provide a new direction for improving the textural properties of plant-based meat by using binders and forming fibrous structures.

## 1. Introduction

The consumption of plant-based protein foods has increased over the years owing to rising health concerns, demand for sustainable food, religious ideologies, and environmental protection [1,2]. As veganism has become a trend, animal protein is being replaced with plant protein, and extensive research has been conducted on plant-based meat. Plant-based meat, also called a meat substitute, simulates the aesthetic qualities and chemical characteristics of traditional meat products [3]. Plant-based meat products in the market are similar in quality (texture and taste) to traditional meat and are based on soy protein [2]. However, due to differences in appearance, texture, taste, and flavor, it is still difficult to fulfill consumer preferences for plant-based meat [4]. The most discussed problem with plant-based meat is the lack of textural properties, which can be solved by adding food additives and modifying the manufacturing conditions or processes [5,6].

In meat science, the term “binding” has several different meanings: water holding capacity (WHC), emulsification capacity, or sticking together meat pieces [7]. Various types of binders, such as egg white [8], cereal [9], carrageenan, transglutaminase, isolated soy protein, and wheat fiber [10], are used alone or in mixture in meat products. Non-meat ingredients are used as binders in meat products to improve texture, color, flavor, and processing quality [11,12]. Binders are also used for improving the nutritional value of meat products by acting as fat replacers [13] and dietary fiber suppliers [14] and extending shelf life [15]. In recent years, binders have been used for the texturization of cultured meat [16] and plant-based meat [17].

In this study, binding or binder refers to the bonding of plant proteins or bonding caused by additives. Plant proteins used for plant-based meat are usually texturized via an extrusion process to provide protein quality similar to that of animal protein [1,18]. Textured vegetable protein (TVP) has less fat and lacks binding capacity and elasticity when forming a gel; it generally uses binders in the manufacture of pulverized products [19]. Many studies have been conducted on binders suitable for animal proteins; however, studies on binders suitable for plant proteins for manufacturing plant-based meat are still insufficient. Therefore, there is a need to study suitable binders for plant proteins, especially soy proteins, which are the most commonly used raw materials for plant-based meat.

Transglutaminase (TG) is an enzyme that catalyzes the formation of the cross-linking isopeptide bond between the ε-amino group of lysine and the γ-carboxyamide group glutamine residues in proteins [20]. It is used in the food industry for the texturization of fish, meat, and dairy products such as surimi, fish ball, ham, cheese, and yogurt. Many studies have reported that TG is an enzyme suitable for forming cross-links with plant proteins, such as soybean, wheat, rice, pea, lupine, sunflower, and sesame [21,22,23]. Qin et al. [24] reported that the rheological properties of SPI gels were improved by the addition of TG, forming a denser and more homogeneous gelation network. Additionally, TG improves the textural properties of plant-based meat by influencing the physical properties of plant protein networks [16,23,25].

Glucono-δ-lactone (GdL) is a coagulant mainly used for tofu production. GdL decreases pH by progressively hydrolyzing and accumulating gluconic acid [26]. When the pH is lowered below the isoelectric point in an aqueous solution saturated with protein, coagulation of the protein occurs, forming a protein gel [27]. GdL has been used in tofu manufacturing and acid-induced gelation of soy proteins [28]. Although GdL has rarely been applied to plant-based meat, due to its positive effect on soy protein gelation, it is suggested to improve the physical properties of plant-based meat. Hui et al. [29] reported transglutaminase and glucono-δ-lactone-treated tofu gel shows a slow digestion rate, and it has more chances to release bioactive peptides than soymilk. 

The textural properties of plant-based meat are suggested to differ according to different protein gelation structures induced by each binder. Thus, in this study, two binders were applied alone or in combination to obtain plant-based meat patties (PBMPs). This study aimed to investigate the effects of different binders on the textural properties and protein matrix structure of PBMPs.

## 2. Materials and Methods

### 2.1. Materials

Textured vegetable protein (TVP, SUPRO MAX 5050; DuPont Korea, Seoul, Korea) and soy protein isolate (ISP, Avention, Incheon, Korea) were selected as ingredients for PBMPs. Gd (Sigma-Aldrich, Burlington, MA, USA) and TG (AJINOMOTO, Tokyo, Japan), which have 98 units/g enzyme activity, were used as binders. Canola oil (Haepyo, Seoul, Korea) was used for the emulsion added as an animal fat substitute.

### 2.2. Preparation of PBMPs

**Figure 1:**
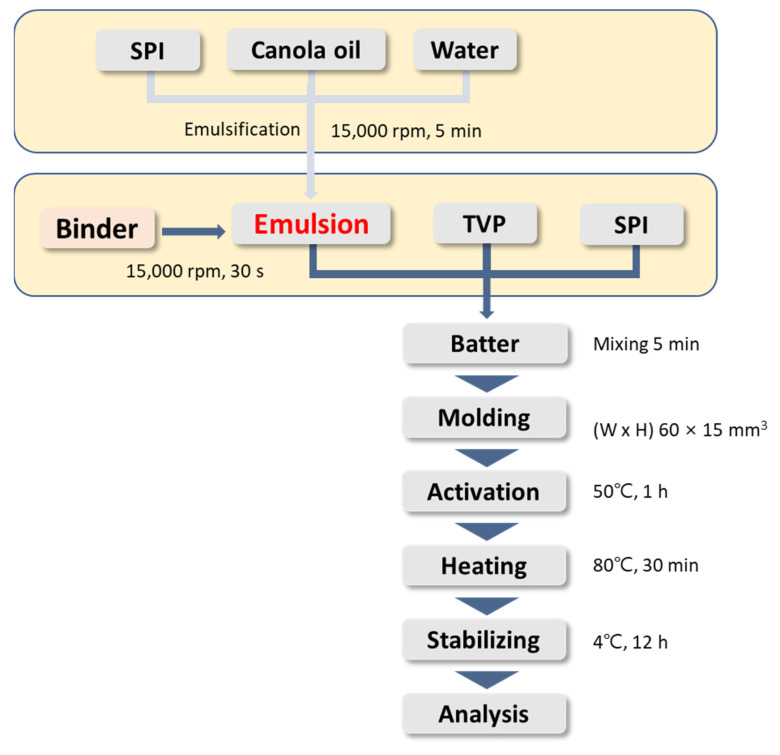
Flow diagram for the manufacture of plant-based meat patties (PBMPs) with different binders.

TVP was immersed in 10 volumes of distilled water for 2 h, and excessive exudates were removed using a centrifugal dehydrator (WS-6600; Hanil Electric, Seoul, Korea) for 5 min at 1200 rpm and used as a base ingredient for PBMPs. The emulsion was prepared by mixing 1% (*w*/*w*) SPI solution with canola oil in a 6:4 (*v*/*v*) ratio and homogenizing at 15,000 rpm for 3 min using a high-speed homogenizer (T25 digital ULTRA-TURRAX; IKA, Staufen, Germany). GdL and TG were mixed in the prepared emulsions at ratios of 10:0, 7:3, 5:5, 3:7, and 0:10, and homogenized at 15,000 rpm for 30 s. All ingredients were mixed as shown in Table 1 and blended using a food blender for 5 min (550 W, Multiquick 3 Vario; Braun, Kronberg im Taunus, Germany). Then, 30 ± 1 g of batter was molded into a cylindrical shape (60 × 15 mm) and left in a 50 °C incubator (HL43; SANGWOO, Bucheon-si, Gyeonggi-do, Korea) for 1 h to activate TG. The samples were then heated in a water bath at 80 °C for 30 min and cooled to room temperature. The samples were stored at 4 °C for 12 h prior to the analysis (Figure 1).

### 2.3. Visible Appearance

The external and internal appearances of plant-based meat were observed using a camera (EOS 100D; Canon, Tokyo, Japan).

### 2.4. pH Measurements

The pH values of the GdL solution and the PBMP were determined using a pH meter (S220, Mettler Toledo GmbH, Greifensee, Switzerland). The GdL solution was prepared by the same concentration and heat process as PBMP. PBMP for pH measurement was prepared without oil and diluted 10 times with deionized water before measuring.

### 2.5. Color Analysis

The color of PBMPs was measured using a colorimeter (CR-400; KONICA MINOLTA, Tokyo, Japan; illuminant D 65, 2° observer angle, 8 mm aperture diameter, and measurement geometry d/0) operating on the CIE system to measure the parameters L* (lightness), a* (redness), and b* (yellowness). Before use, the colorimeter was standardized using a white calibration plate (CR-A44, Konica Minolta Sensing Inc., Osaka, Japan, L* = 96.06, a* = −0.38, b* = 1.23). Each measurement was performed in triplicate and the average value was recorded.

### 2.6. Cooking Loss (CL) and Water Holding Capacity (WHC)

CL of PBMP samples was calculated by measuring the weight of each sample before and after cooking, following the method of Forghani et al. [23]. The CL was calculated using Equation (1). The experiment was repeated five times for each sample.

(1) $$Cooking\;loss\left(\%\right)=\frac{weight\;before\;cooking\left(g\right)-weight\;after\;cooking\left(g\right)}{weight\;before\;cooking\left(g\right)}\times 100$$

The WHC of PBMPS was measured by modifying the method described by Bastos et al. [30]. The cooked sample (1 ± 1 g) was wrapped with filter paper (Whatman no. 1) and placed in a 15 mL conical tube. The tube was then centrifuged at 3000 rpm at 25 °C for 20 min (LaboGene 1736R; GYROGEN, Daejeon, Korea). The weights of the samples before and after centrifugation were measured, and the WHC was calculated using Equation (2). The experiment was repeated five times for each sample.

(2) $$Water\;holding\;capacity\;\left(\%\right)=\frac{weight\;before\;centrifugation\left(g\right)}{weight\;after\;centrifugation\left(g\right)}\times 100$$

### 2.7. Mechanical Properties

The mechanical properties of PBMPs were measured according to the method suggested by Forghani et al. [23], with slight modification, where the samples were equilibrated to room temperature and formed in a block-shaped cube (2 × 2 × 1.5 cm). The mechanical properties were measured using a texture analyzer (CT3; Brookfield Engineering Labs Inc., Middleborough, MA, USA). The hardness of PBMPs was measured using a cutting-shearing test. A TA-SBA-13 probe was used, and the analysis conditions were a trigger load of 10 g, a compression distance of 30 mm, and a test speed of 2.5 mm/s. Adhesiveness, cohesiveness, springiness, gumminess, and chewiness were measured via texture profile analysis (TPA) of the central portion of PBMPs. A cylindrical probe (TA4/1000, diameter 38.1 mm) was used, and the analysis conditions were 40% deformation, 10 g trigger load, and test speed of 2.5 mm/s. The measurements of each sample were repeated twenty times or more and expressed as the average and standard deviation values.

### 2.8. Microstructure

The microstructure was observed after preparing the samples using the method described by Samard & Ryu [31]. Briefly, PBMP samples were cut into vertical thin slices and frozen at −100 °C for 24 h in a deep freezer (CLN; NIHON-FREEZER, Tokyo, Japan). Frozen PBMP samples were dried in a freeze dryer (FDCF-12012; Operon, Gimpo-si, Gyeonggi-do, Korea) at a pressure of 5 Pa and a temperature of −80 °C for 48 h. Micrographs of the samples were taken at ×200 magnification using a scanning electron microscope (TM4000Plus; Hitachi, Tokyo, Japan) with an accelerating voltage of 15 kV.

### 2.9. Sensory Evaluation

The sensory test was conducted on ten experienced graduate students who had received prior training. PBMP samples were provided at the same temperature (25 °C) and size (1 × 1 × 0.5 cm) after a 3-digit random number was assigned to each sample. The test was conducted using a seven-point scoring test [32]: color, flavor, hardness, elasticity, compactness, juiciness, meat similarity, and overall acceptance. Before the test, the procedure for sensory evaluation was approved by the institutional review board (approval no. 700355-201901-HR-294). Written consent was obtained from all the participants before conducting sensory evaluation.

### 2.10. Statistical Analysis

All experiments were performed using the SPSS Statistics software (ver. 24.0; SPSS Inc., Chicago, IL, USA), except for sensory evaluation. The significance of the results was analyzed using one-way analysis of variance and Duncan’s multiple range test, which were conducted at *p* < 0.05, to verify the statistical significance of each sample.

## 3. Results and Discussion

### 3.1. Visible Appearance

**Figure 2:**
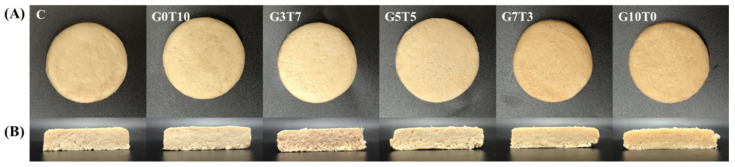
External (**A**) and internal (**B**) appearance of plant-based meat patties (PBMPs) with different binders. C, control; G0T10, GdL:TG = 0:10; G3T7, GdL:TG = 3:7; G5T5, GdL:TG = 5:5; G7T3, GdL:TG = 7:3; G10T0, GdL:TG = 10:0.

The external and cross-sectional appearances of the plant-based meat after cooking are shown in Figure 2. The addition of TG made it brighter than the control, and the shape of PBMPs, such as diameter or height, before cooking was best maintained compared to the other samples. However, the addition of GdL resulted in a darker and yellow color compared to the control, and it remarkably increased as the concentration of GdL was increased. The addition of TG to PBMPs seems to increase the brightness due to cross-linking between proteins and the formation of protein aggregates, while the addition of GdL seems to induce protein denaturation by increasing the acidity and the Maillard reaction between denatured protein and free sugar, thereby affecting the decrease in the brightness of PBMPs [33,34,35]. In addition, compared to the shape of the patty before cooking, many deformations occurred, and it became flat. The addition of TG or GdL alone resulted in a smoothly cut cross-section; when TG and GdL were added as a mixture, the cross-section appeared to be roughly cut. However, the binding force did not decrease, or pores were not formed (G7T3). Therefore, the addition of TG and GdL mixture seemed to better form a fibrous structure on the PBMPs.

### 3.2. pH of GdL Solution and PBPs

**Figure 3:**
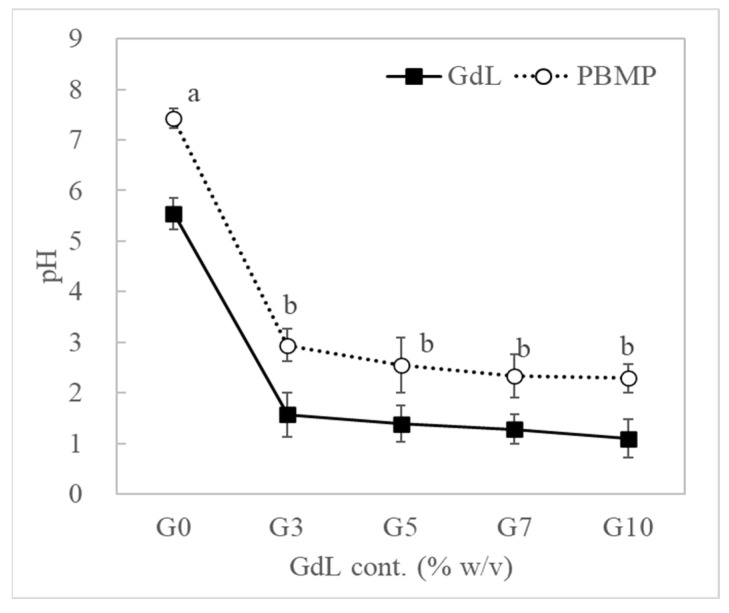
Comparison of pH value of GdL solution and PBMP depends on the GdL concentration.

The pH of the GdL solution and PBMP are shown in Figure 3. In order to understand the physical properties of PBMP affected by GdL concentration, the pH of the GdL solution and PBMP was measured. While the pH of the GdL solution (G3~G10) was in the range of pH 1.10 to 1.57, the PBMP (G3~G10) was in the range of 2.29 to 2.95. The addition of SPI and TVP seemed to have an effect on the increase in pH. As the amount of GDL increased, the pH of the GdL solution and PBMP was decreased, but no significant difference was observed depending on the concentration used (*p* > 0.05). The lower GdL concentration of PBMP is closer to the protein isoelectric point (≈pH 4.6). In addition, the optimal activation pH of TG is pH 5.0~8.0 [36], but the pH of the PBMP was in the pH range of 2.29–2.95. Therefore, the concentration of GdL has a major influence on the physical properties of PBMP, and protein seemed more texturized with low GdL (G3T7), followed by high GdL PBMP(G10T0). Meanwhile, high GdL induced protein denaturation and conditions under which the Maillard reaction can easily occur [33,35].

### 3.3. Color

Color is an important characteristic that affects consumer acceptance in the food industry [8]. Table 2 shows the color change after cooking plant-based meat with binders. The L* value of the control was 69.81, while those of G0T10 and G3T7 increased to 71.26 and 74.3, respectively (*p* < 0.05). Changes in L*, a*, and b* values indicate changes in the protein particles [28]. As the cross-linking between proteins increased with the addition of TG, the protein aggregates increased, which may have increased the brightness. In contrast, the addition of GdL significantly decreased the L* value and increased the a* and b* values of the plant-based meat. The L* values of G5T5, G7T3, and G10T0 were 73.67, 64.30, and 58.98, respectively. The addition of GdL was thought to affect the color change of plant-based meat by changing the reflective properties owing to protein aggregates. As the GdL concentration increased, the acidiy of the plant-based meat dough increased, which could denature the protein in the dough. In the unfolded protein structure, it may have reduced the L* value and increased the a* and b* values of plant-based meat via the Maillard reaction with the glass sugar of TVP [33]. These results suggest that GdL may have led to the browning of the non-enzyme Maillard to protein aggregates [35].

### 3.4. Cooking Loss (CL) and Water Holding Capacity (WHC)

**Figure 4:**
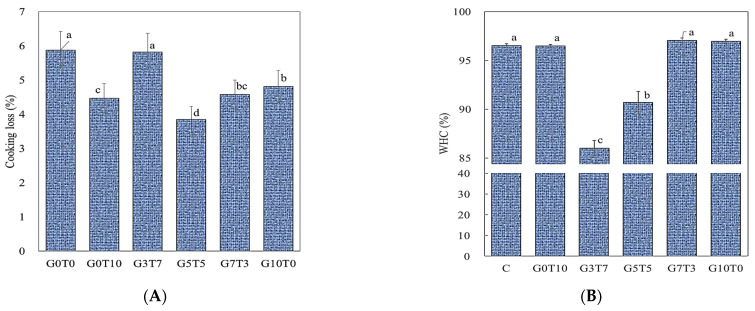
Cooking loss (**A**) and water holding capacity (**B**) of PBMPs depending on the ratio of binders. C, control; G0T10, GdL:TG = 0:10; G3T7, GdL:TG = 3:7; G5T5, GdL:TG = 5:5; G7T3, GdL:TG = 7:3; G10T0, GdL:TG = 10:0. a–c indicate that different superscripts in a column are statistically significant differences (*p* < 0.05).

An important characteristic of patties and other meat products is their ability to retain water and other liquid components before and after heat treatment [37]. In this study, CL was used to measure the moisture lost during heat treatment, and the WHC was used to measure the stability of plant-based meat during storage. The differences in CL and WHC of plant-based meat with added binder are shown in Figure 4. The CL of the control was 6.13%, and compared with the control, the CL was significantly reduced to 4.97 and 5.12%, respectively, with the addition of TG and GdL alone (*p* < 0.05). When TG and GdL were mixed and added, G5T5 and G7T3 showed the lowest CL values at 4.16 and 4.81%, respectively (*p* < 0.05). In contrast, the CL of G3T7 cells was 6.00%, which was similar to that of the control, and the mixed ratio of TG and GdL did not have a positive effect on CL reduction. Hence, the addition of a binder did not have a significant effect on texture improvement (*p* > 0.05). 

The WHC of the control was 96.53%, indicating that the binding force between the protein and water was already high (*p* < 0.05). G0T10, G7T3, and G10T0 showed similar values to the control, while G5T5 and G3T7 showed a decrease of 90.69% and 86.02%, respectively (*p* < 0.05). These results were obtained due to the interaction of the two binders. Zhang et al. [38] reported that GdL reaches an appropriate pH and induces gel formation by properly changing the cohesive structure of proteins for cross-linking by TG, suggesting the synergistic effects of TG and GdL. However, excessive GdL may have degraded the activation of TG by dropping the pH above an appropriate value. In addition, G3T7 has the lowest WHC even though it has the highest hardness, which shows that soft gels have better WHC than hard gels [39]. In conclusion, this result showed that the addition of binders did not increase the water content and WHC of cooked plant-based meat, but generally decreased the total CL.

### 3.5. Mechanical Properties

The texture of plant-based meat is an essential factor for mimicking the muscle texture of actual meat and is an important characteristic in the development of plant-based meat products because it affects consumer product selection [40]. Table 3 shows the measurement results of the mechanical properties of plant-based meat with the addition of binders. The cutting–shearing test revealed that the addition of TG and GdL had significant effects on the texture parameters of plant-based meat (*p* < 0.05). The hardness of the control was 5.64 N, the TG-added sample increased to 7.61 N, and the GdL-added sample improved to 13.56 N (*p* < 0.05). With the addition of TG, the springiness of plant-based meat increased from 2.19 to 3.71 mm, gumminess improved significantly from 6.40 to 13.07 N, and chewiness from 12.88 to 53.63 mJ (*p* < 0.05), compared to the control. According to Forghani et al. [23], the addition of TG to formulations enhances the hardness and elasticity of food by inducing new cross-linking in the protein structure and enhancing the cohesiveness of batters. Lee and Hong [17] reported that TG improved all TPA parameters of soy patties. The springiness of the GdL sample was 1.33 mm, which was lower than that of the control. GdL had a negative effect on gumminess and chewiness (*p* < 0.05). In this study, a positive synergistic interaction effect was observed when the binder was added as a mixture rather than when it was added alone. Qin et al. [24] reported that the enzymatic reaction of TG can be accelerated by modifying the fold structure of the protein with acid pretreatment. Herz et al. [26] reported that the combination of slow acidification and cross-link formation when simultaneously adding TG and GdL can produce acidic gels with improved textural properties and shelf life. Accordingly, a stronger protein gel was obtained in the sample to which the binder was added simultaneously, and the hardness was improved in plant-based meat. The improved hardness and chewiness are thought to have a positive effect on quality by imparting desirable texture to plant-based meat lacking chewing texture.

### 3.6. Microstructure

**Figure 5:**
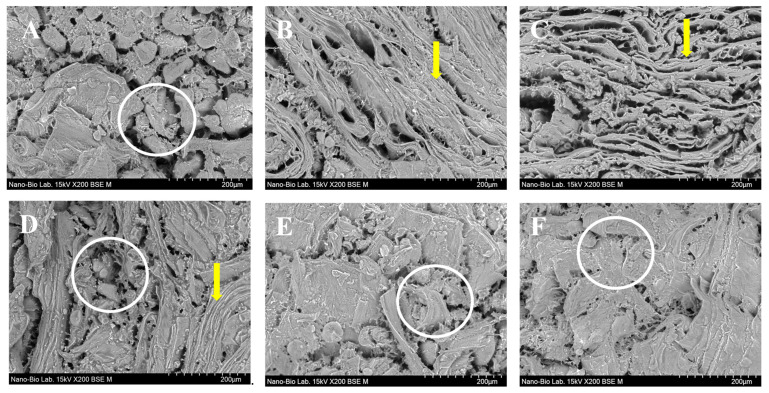
Scanning electron microscope images of vertically cut PBMPs with different binders (×200 magnification). (**A**): control; (**B**): G0T10, GdL:TG = 0:10; (**C**): G3T7, GdL:TG = 3:7; (**D**): G5T5, GdL:TG = 5:5; (**E**): G7T3, GdL:TG = 7:3; (**F**): G10T0, GdL:TG = 10:0. The regions indicated by circles and arrows represent the observed protein aggregates and fiber structure, respectively.

To determine the cause of the change in the WHC and hardness with the addition of the binder, the binder-added plant-based meat was cut vertically and its microstructure was observed. Scanning electron microscopy images are shown in Figure 5, and the regions indicated by circles and arrows represent the observed protein aggregates and fiber structure, respectively. In the control, uniform protein aggregation and wide pores between aggregated proteins were observed (Figure 5A). With the addition of binders, TG and GdL induced changes in the protein structure of plant-based meat via different mechanisms. In G0T10, a linear fibrous structure was formed by TG, and the pores between the protein matrices became narrower than those in the control, and the structure was uniform and more distinct (Figure 5B,C). This was the basis for the higher hardness values of G3T7 than G0T10 in the previous TPA results, which suggests a close relationship between the mechanical properties and microstructure. In G0T10 and G7T3, the fibrous structure induced by TG was not observed. GdL catalyzed protein aggregation and formed a denser protein structure (Figure 5E,F). In G5T5, the fibrous structure induced by TG and the protein aggregation catalyzed by GdL were simultaneously observed (Figure 5D). A crucial property for plant-based meat products to mimic actual meat is the fibrous structure of the muscle tissue, which provides texture [1]. Erdem et al. [41] reported that with the addition of TG, meatballs had a firmer and more regular gel structure. Therefore, G0T10 and G3T7 with a fibrous structure can produce a texture similar to that of actual meat, which can have a positive effect on the sensory characteristics of plant-based meat.

### 3.7. Sensory Evaluation

The sensory test results for plant-based meat with added binders are shown in Table 4 and Table 5. There were significant differences in the flavor, hardness, elasticity, compactness, juiciness, meat similarity, and overall preference based on the type and mixing ratio of the added binder. As a result of the intensity test, the control recorded a low score of 2.61, similar to meat, owing to low hardness, elasticity, and lack of juiciness. However, with the addition of TG, hardness, elasticity, compactness, and juiciness were increased and scored high points. Consequently, G0T10 received high scores for all test items, and the overall preference was the highest at 4.11 points. G3T7 was found to have the highest hardness in the mechanical property analysis. In addition, the juiciness was improved by the binder compared to the control in the intensity test. According to Kim et al. [42], juiciness is related to the CL and WHC of meat products. It was predicted that the previously analyzed results of the WHC and sensory preference would show a similar trend. However, regardless of the WHC, juiciness was observed in the plant-based meat with TG and GdL mixtures in the sensory test results. It was found that the addition of binders, especially TG and GdL as a mixture, generally improved the elasticity and hard texture. However, the overall preference for improved hardness was not high compared with that of the control.

## 4. Conclusions

**Figure 6:**
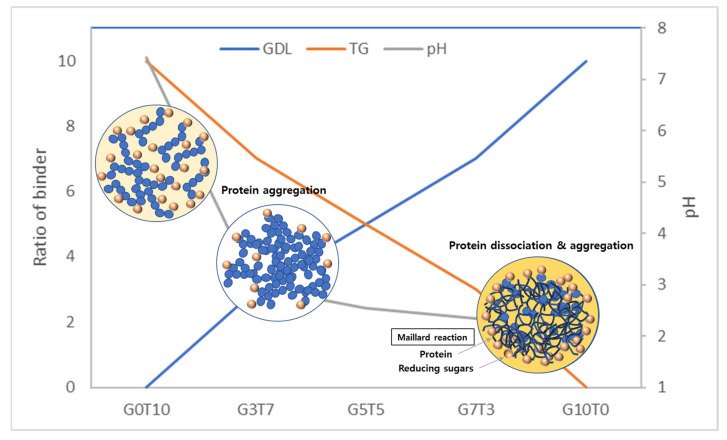
Color and structure change of soy protein depends on the binder ratio.

In this study, two types of binders were used to improve the quality of plant-based meat, and their effects on the physicochemical and textural properties of plant-based meat were determined. TG improves hardness by forming a fibrous structure in plant-based meat while also improving the gumminess and chewiness with increased springiness. GdL improved the hardness of plant-based meat by inducing protein aggregation. The addition of binders individually resulted in reduced CL and a high WHC of plant-based meat, and the addition of the binder had positive effects on the physicochemical and textural properties of the plant-based meat (Figure 6). The addition of TG and GdL as a mixture had a synergistic effect on the hardness increase in TPA, which compensated for the insufficient chewability of plant-based meat. In plant-based meat, mimicking the muscle texture of meat is the most important factor; thus, the addition of TG was effective in improving the texture of PBMPs. The texture characteristics can be further improved by adjusting the mixing ratio of TG and GdL via detailed optimal ratio experiments in the future. The results of this study suggest a new direction for improving the textural properties of plant-based meat and forming fibrous structures.

## Figures and Tables

**Table 1:** Formulation ratio of ingredients for plant-based meat with different binders.

Treatments *	Base Ingredients (%)		Emulsion (%)		Binder (%) **	
	TVP ^1)^	SPI ^2)^	DW	Oil	GdL ^3)^	TG ^4)^
Control	65	5.3	17.7	12	-	-
G0T10	65	5.3	17.7	12	-	1
G3T7	65	5.3	17.7	12	3	0.7
G5T5	65	5.3	17.7	12	5	0.5
G7T3	65	5.3	17.7	12	7	0.3
G10T0	65	5.3	17.7	12	10	-

^1)^ Textured vegetable protein with 65% moisture content, ^2)^ soy protein isolate, ^3)^ glucono-δ-lactone, and ^4)^ transglutaminase. * Ratio between GdL and TG, GdL:TG = 0:10, 3:7, 5:5, 7:3, and 10:0. ** Ratio from total weight of base ingredients and emulsion.

**Table 2:** Color of plant-based meat patties (PBMPs) with different binders.

Treatments	L*	a*	b*
Control	69.81 ± 1.15 ^b^	2.50 ± 0.41 ^c^	17.01 ± 0.62 ^b^
G0T10	71.26 ± 1.32 ^b^	2.42 ± 0.14 ^cd^	17.05 ± 0.59 ^b^
G3T7	74.30 ± 0.74 ^a^	2.13 ± 0.11 ^d^	16.38 ± 0.51 ^b^
G5T5	73.67 ± 0.94 ^a^	2.55 ± 0.31 ^c^	16.99 ± 0.81 ^b^
G7T3	64.30 ± 1.51 ^c^	3.10 ± 0.27 ^b^	17.96 ± 0.38 ^a^
G10T0	58.98 ± 1.84 ^d^	3.62 ± 0.19 ^a^	17.99 ± 0.33 ^a^

G0T10, GdL:TG = 0.10; G3T7, GdL:TG = 3:7; G5T5, GdL:TG = 5:5; G7T3, GdL:TG = 7:3; G10T0, GdL:TG = 10:0. ^a–d^ indicate that different superscripts in a column are statistically significant differences (*p* < 0.05).

**Table 3:** Mechanical properties of plant-based meat with different binders.

Treatments *	Hardness ^1)^ (N)	Adhesiveness ^2)^ (mJ)	Cohesiveness	Springiness (mm)	Gumminess (N)	Chewiness (mJ)
Control	8.31 ± 2.11 ^d^	5.53 ± 2.04 ^a^	0.13 ± 0.03 ^c^	1.56 ± 0.26 ^cd^	5.98 ± 0.62 ^e^	9.96 ± 2.56 ^d^
G0T10	10.54 ± 3.17 ^d^	0.58 ± 0.37 ^c^	0.16 ± 0.01 ^b^	3.66 ± 0.25 ^a^	11.33 ± 1.60 ^b^	43.67 ± 9.10 ^b^
G3T7	25.49 ± 6.82 ^a^	1.37 ± 1.00 ^c^	0.21 ± 0.30 ^a^	3.70 ± 0.61 ^a^	15.99 ± 1.35 ^a^	57.76 ± 6.42 ^a^
G5T5	19.18 ± 5.32 ^b^	3.49 ± 0.60 ^b^	0.11 ± 0.05 ^d^	1.75 ± 0.13 ^bc^	6.33 ± 0.78 ^e^	11.53 ± 0.59 ^d^
G7T3	20.80 ± 6.86 ^b^	3.31 ± 1.13 ^b^	0.09 ± 0.17 ^e^	1.37 ± 0.19 ^d^	7.73 ± 0.71 ^d^	11.36 ± 2.61 ^d^
G10T0	14.98 ± 1.32 ^c^	5.83 ± 2.77 ^a^	0.11 ± 0.16 ^d^	1.88 ± 0.42 ^b^	9.49 ± 0.99 ^c^	18.00 ± 4.77 ^c^

^1)^ Analyzed using cutting–shearing test. ^2)^ Analyzed using 2-bite compression test. * G0T10, GdL:TG = 0:10; G3T7, GdL:TG = 3:7; G5T5, GdL:TG = 5:5; G7T3, GdL:TG = 7:3; G10T0, GdL:TG = 10:0. ^a–d^ indicate that different superscripts in a column are statistically significant differences (*p <* 0.05).

**Table 4:** Intensity of sensory properties of PBMPs with different binders.

Treatments *	Color	Flavor	Hardness	Elasticity	Compactness	Juiciness	Meat Similarity
Control	4.11 ± 1.15 ^abc^	3.50 ± 1.54 ^ND^	3.68 ±1.67 ^ND^	3.79 ±1.23 ^ND^	3.89 ± 1.15 ^ND^	2.84 ± 1.17 ^ND^	2.61 ± 1.14 ^ab^
G0T10	3.79 ± 0.85 ^bc^	3.67 ± 1.41	4.21 ± 1.44	3.89 ± 1.20	4.16 ± 1.21	3.00 ± 1.33	3.32 ± 1.20 ^a^
G3T7	3.42 ± 1.02 ^c^	3.67 ± 1.57	4.53 ± 1.07	3.47 ± 1.07	3.58 ± 1.26	3.05 ± 0.85	3.05 ± 1.22 ^a^
G5T5	3.53 ± 1.02 ^c^	3.61 ± 1.58	4.00 ± 1.15	4.05 ± 1.39	4.16 ± 1.26	3.32 ± 1.20	3.37 ± 1.26 ^a^
G7T3	4.47 ± 1.22 ^abc^	4.06 ± 1.86	4.42 ± 1.17	3.47 ± 1.47	3.79 ± 1.03	3.21 ± 1.03	2.67 ± 1.46 ^ab^
G10T0	4.74 ± 1.56 ^a^	4.11 ± 1.32	3.63 ± 1.46	3.21 ± 1.32	3.68 ± 1.34	2.68 ± 1.34	2.00 ± 0.94 ^b^

* G0T10, GdL:TG = 0:10; G3T7, GdL:TG = 3:7; G5T5, GdL:TG = 5:5; G7T3, GdL:TG = 7:3; G10T0, GdL:TG = 10:0. ^a–c^ indicate that different superscripts in a column are statistically significant differences (*p* < 0.05), ^ND^ indicates statistically no significant differences (*p* < 0.05).

**Table 5:** Preference of sensory properties of PBMPs with different binders.

Treatments *	Color	Flavor	Hardness	Elasticity	Compactness	Juiciness	Overall Acceptance
Control	4.21 ± 1.23 ^ND^	4.11 ± 1.60 ^a^	4.37 ± 1.42 ^a^	3.68 ± 1.34 ^ab^	3.74 ± 1.33 ^ab^	3.21 ± 1.51 ^ND^	3.53 ± 1.39 ^ab^
G0T10	4.26 ± 1.15	4.33 ± 1.33 ^a^	4.26 ± 1.37 ^a^	4.05 ± 1.39 ^ab^	4.26 ± 1.37 ^a^	3.42 ± 1.50	4.11 ± 1.70 ^a^
G3T7	3.63 ± 1.54	2.83 ± 1.34 ^b^	3.26 ± 1.15 ^b^	3.32 ± 1.11 ^bc^	3.42 ± 1.22 ^ab^	3.00 ± 1.25	2.74 ± 1.19 ^bcd^
G5T5	3.89 ± 1.10	2.67 ± 1.08 ^b^	3.84 ± 1.12 ^ab^	4.26 ± 1.24 ^a^	3.89 ± 1.18 ^ab^	3.32 ± 1.29	3.00 ± 1.49 ^bc^
G7T3	4.11 ± 1.49	2.28 ± 1.07 ^b^	3.74 ± 1.19 ^ab^	3.68 ± 1.34 ^ab^	3.84 ± 1.07 ^ab^	3.05 ± 1.22	2.22 ± 1.17 ^cd^
G10T0	4.16 ± 1.50	2.83 ± 1.34 ^b^	3.53 ± 1.50 ^ab^	2.74 ± 1.15 ^c^	3.21 ± 1.27 ^b^	2.63 ± 1.42	2.00 ± 1.05 ^d^

* G0T10, GdL:TG = 0:10; G3T7, GdL:TG = 3:7; G5T5, GdL:TG = 5:5; G7T3, GdL:TG = 7:3; G10T0, GdL:TG = 10:0. ^a–d^ indicate that different superscripts in a column are statistically significant differences (*p* < 0.05), ^ND^ indicates statistically no significant differences (*p* < 0.05).

## Data Availability

The datasets generated in this study are available upon request from the corresponding author.
